# Correction: Naringin protects endothelial cells from apoptosis and inflammation by regulating the Hippo-YAP Pathway

**DOI:** 10.1042/BSR-20193431_COR

**Published:** 2020-07-30

**Keywords:** Naringin, Endothelial cells, apoptosis, inflammation, Hippo-YAP Pathway

The original article “Naringin protects endothelial cells from apoptosis and inflammation by regulating the Hippo-YAP Pathway” (*Biosci Rep* (2019) **40**(3), DOI: 10.1042/BSR20193431) contained an image error and an incorrect description of the data presented. The authors state that they had placed a repeated plot in Figure 2C, and erroneously presented the data in the manuscript as mean±SD, rather than the intended mean±SEM. The corrected figure and its legend is presented below. The authors confirm that the correct equation was used in their analyses, and that this correction does not change their results.

**Figure 2:**
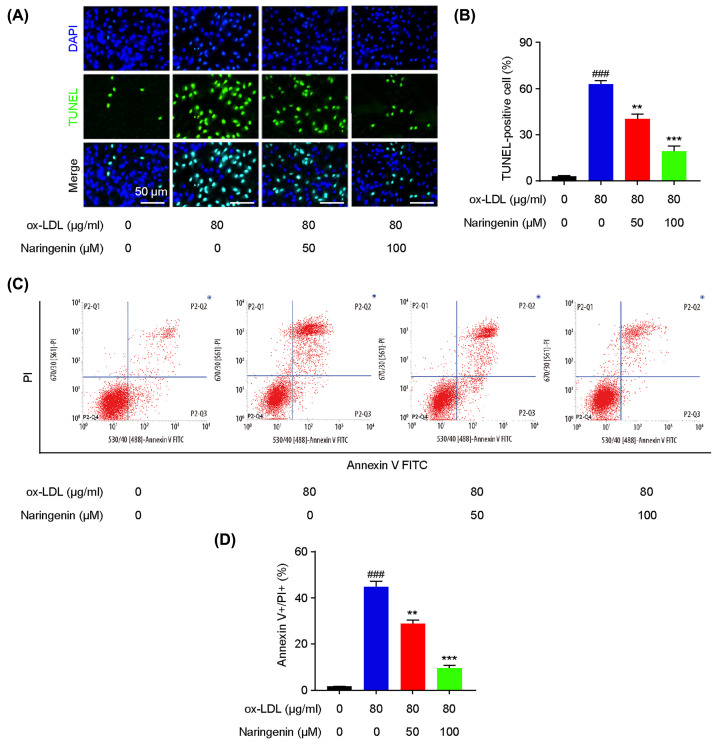
Naringin attenuates ox-LDL-induced apoptosis in HUVECs Naringin could inhibit ox-LDL stimulated endothelial apoptosis. HUVECs were pretreated with (50 or 100 µM) naringin for 2 h, followed by treatment with 80 µg/l ox-LDL for 24 h. (**A**) Representative image of TUNEL assay. (**B**) The percentage of TUNEL-positive cells was quantified. (**C**) Apoptosis of HUVECs in different groups was examined with a FITC Annexin V apoptosis kit, via flow cytometry. (**D**) Quantitative data show that ox-LDL increased the apoptotic rate in HUVECs, which was significantly dose-dependently reversed by naringin. Data are presented as mean±SEM, *n* = 6, ^###^*P*<0.001 vs. the control group, **P<0.01 and ***P<0.001 vs. the ox-LDL group.

